# An empirical comparison of different implicit measures to predict consumer choice

**DOI:** 10.1371/journal.pone.0183937

**Published:** 2017-08-25

**Authors:** Oliver Genschow, Jelle Demanet, Lea Hersche, Marcel Brass

**Affiliations:** 1 Social Cognition Center Cologne, University of Cologne, Cologne, Germany; 2 Department of Experimental Psychology, Ghent University, Ghent, Belgium; 3 Department of Psychology, University of Basel, Basel, Switzerland; University of Bologna, ITALY

## Abstract

While past research has found that implicit measures are good predictors of affectively driven, but not cognitively driven, behavior it has not yet been tested which implicit measures best predict behavior. By implementing a consumer context, in the present experiment, we assessed two explicit measures (i.e. self-reported habit and tastiness) and three implicit measures (i.e. manikin task, affective priming, ID-EAST) in order to test the predictive validity of affectively versus cognitively driven choices. The results indicate that irrespective of whether participants chose affectively or cognitively, both explicit measures, but not the implicit measures, predicted consumer choice very strongly. Likewise, when comparing the predictive validity among all measures, the explicit measures were the best predictors of consumer choice. Theoretical implications and limitations of the study are discussed.

## Introduction

The main goal of psychological research is to predict individuals’ future behavior. In order to predict a person’s future, researchers have focused on the assessment of explicit as well as implicit attitudes (for overviews, see [1,2]). Explicit attitudes refer to the construct social psychologists commonly try to assess by means of questionnaires or interviews. For implicit attitudes different tasks and definitions have been proposed. For the present purpose, implicit attitudes can be defined as evaluative responses toward an object, which, in contrast to explicit attitudes, are not necessarily subject to introspection ([1] for a discussion of the term implicit see [3]). To measure such implicit attitudes, researchers within the last decades have developed different tasks. Thereby, three classes of measures can be differentiated: affective priming tasks, implicit associations tests and approach-avoidance tasks.

### Implicit measures

#### Affective priming

One of the first and most often used implicit measures is the affective priming task [4,5]. Within this task, participants categorize target stimuli as being positive or negative. Each target stimulus is preceded by a prime. The core idea underlying an affective priming task is that targets sharing the valence of the prime lead to faster categorizations than targets that do not share the valence of a prime. For instance, in order to measure a person’s attitude toward chocolate and fruit, one can present on each trial the picture of a chocolate or a fruit as prime stimulus followed by a positive or a negative target stimulus that participants categorize as being positive or negative in valence. If chocolate facilitates responding to positive relative to negative target stimuli, this effect would indicate a positive attitude toward chocolate.

#### Implicit association tests

During a typical Implicit Association Test (IAT; [6]), participants see stimuli that belong to one of four categories (e.g., positive, negative, chocolate, fruit) and are asked to categorize each stimulus by pressing one of two keys. Two of the four categories (e.g., positive and chocolate) are assigned to the first key, and the two other categories (e.g., negative and fruit) are assigned to the second key. The core idea underlying the IAT is that responses should be speeded up when the two categories of a response are strongly associated with each other. For instance, if a person perceives chocolate very positive, she or he should be faster in responding to chocolate stimuli when the same key has to be pressed for positive stimuli, as compared to negative stimuli.

A task similar to the IAT is the Identification Extrinsic Affective Simon Task (ID-EAST; [7]). Like the IAT, the ID-EAST relies on the principle that it is easier to give a response that is associated with positive valence to positive as compared to negative items and vice versa for responses associated with negative valence. For instance, in order to measure the associations of the concepts chocolate and fruit, stimuli of both categories are presented intermixed with positive and negative stimuli. All stimuli are presented in different colors (e.g., green and blue). Participants are instructed to evaluate all stimuli by pressing one key for positive stimuli and the other key for negative stimuli, irrespective of the color of the stimuli. The function of these trials is to link the responses with positive or negative valence. However, for chocolate and fruit stimuli, participants are asked to respond not on the basis of valence but on the basis of the color. In the case of a positive attitude toward chocolate, individuals are expected to respond faster to chocolate stimuli when the same response key is used to identify positive stimuli, as when it is used to identify negative stimuli. Whereas previous versions of the ID-EAST (i.e. the standard EAST) failed to provide a reliable (split-half reliability) or valid (correlations with explicit ratings) measure of attitudes (for an overview, see [8]), the ID-EAST has been found to provide scores that were fairly reliable and valid (e.g., [9,10,11]). Moreover, the ID-EAST has been found to perform at a level close to that of the IAT while overcoming some of the limitations of the IAT (e.g., the ID-EAST provides a measure of single attitudes; see [12]).

#### Approach-avoidance measures

During the last decade, different approach-avoidance measures have been put forward (e.g., [13,14–18]). The common ground of all these approach-avoidance tasks is the hedonic principle that people approach pleasure and avoid pain (for a review, see [19]). Based on this principle it is assumed that positive items elicit a behavioral approach tendency and negative items elicit an avoidance tendency in individuals. Taking advantage of this principle, Chen and Bargh [15] let participants respond to positive and negative stimuli by pushing or pulling a lever. The results showed that participants were faster at responding to negative stimuli when pushing rather than pulling the lever, but were faster at responding to positive stimuli when pulling rather than pushing the lever. Brendl et al. [14] introduced a slightly different technique. In their studies, they presented participants’ name next to either positive or negative stimuli. Participants then moved the presented stimuli with a joystick as quickly as possible either toward or away from their name. The results showed that participants moved positive stimuli faster toward their name than away from their name and vice versa for negative stimuli. Despite using a joystick to measure approach and avoidance tendencies, other researchers measured finger movements toward or away from a stimulus and found similar results (e.g., [13,17,20]).

While the above-reviewed literature indicates that movements toward oneself represent approach tendencies and movements away from oneself represent avoidance tendencies, other research suggests that it is not the movement per se, but rather the context that gives any movement its meaning (e.g., [18,21,22–24]). In line with this idea, in the manikin task [16], participants press a neutral key on a computer keyboard to let a manikin run toward or away from a stimulus on a screen. In line with other approach-avoidance measures, the common finding is that participants respond faster to positive stimuli when their response makes the manikin move toward the stimuli than away from the stimuli. Conversely, reaction times are faster to negative stimuli when the response makes the manikin walk away from the stimuli as compared to walk toward the stimuli (see also [25,26]). When comparing joystick tasks with the manikin task, Krieglmeyer and Deutsch [25] found that the manikin task is more sensitive to valence and more strongly related to self-report scales than the joystick task.

### Context factors and predictive validity

Millar and Tesser [27,28] assumed that behavior is either driven cognitively or affectively and showed that attitudes measured under an affective focus correlate more strongly with affectively driven behavior than with cognitively driven behavior and vice versa for attitudes measured under a cognitive focus (cf. also [29]). Thereby, it has been found that explicit measures are of particular importance for the prediction of deliberate, controlled and cognitively driven behavior [2,4,30,31]. Conversely, implicit measures are better predictors of impulsive and affectively driven behavior [17,32–37]. For instance, Scarabis, Florack and Gosejohann ([37], see also [38]) assessed the IAT as implicit measure and then let participants chose between a chocolate bar and a fruit while either being in an affective or cognitive focus. The results show that the IAT was a better predictor when participants chose in an affective, as compared to a cognitive focus. When implementing the same focus manipulation and choice options, Genschow and colleagues [17] found the same results for an approach-avoidance measure.

In sum, it is well known that implicit measures better predict affective driven than cognitive driven behavior. But which measure actually predicts behavior the best? Bar-Anan and Nosek [39] compared seven different implicit measures in terms of psychometric qualities of seven indirect attitude measures across three attitude domains (race, politics, and self-esteem). Specifically, they assessed the Implicit Association Test (IAT; [6]), the Brief IAT (BIAT; [40]), the Single-Target IAT (ST-IAT; [41]), the Go–No-Go association task [42], the Affective Misattribution Procedure (AMP; [43]), and the Sorting Paired Features task (SPF; [44]), as well as Evaluative Priming [5] and found overall good psychometric quality. Thereby, the IAT showed highest and the Evaluative Priming lowest quality. Despite this comprehensive comparison of implicit measures, to the best of our knowledge, no study actually compared the predictive validity of different implicit measures against each other. Moreover, while the predictive validity of the IAT is well studied (e.g., [33,35,37,45,46,47]), less is known about the predictive validity of other implicit measures. By implementing a consumer setup, the aim of the present study was, thus, to compare different implicit measures with explicit measures and to test their predictive validity of affectively and cognitively driven choices of chocolate versus fruit. As implicit measures we chose tasks that have been reported to be valid and reliable as well as can be used with pictures as stimuli. That is, we chose a standard affective priming task that has been used previously by Degner and Wentura [48]. For an IAT-like task we chose the ID-EAST, because it has been found to perform at a level close to that of the IAT while overcoming some of the IAT’s limitations [12]. For the approach-avoidance task we applied the manikin task, because it is more sensitive to valence and more strongly related to self-report scales than the joystick tasks [25]. Finally, in order to cross-validate the predictive validity of the implicit measures we assessed also two explicit ratings—namely participants’ self-reported habit and taste of chocolate and fruit.

## Method

### Ethics statement

The study was conducted in accordance with the ethical standards of the 1964 Declaration of Helsinki and approved by the rules of the Institutional Review Board from the Faculty of Psychology and Educational Science of Ghent University. All participants provided informed consent at the beginning of the experiment and were informed that participation was voluntary and that all answers were processed and stored anonymously.

### Data availability statement

The data file of the study is available from the Open Science Framework database. The URL necessary to access our data is: https://osf.io/wnp9k/

### Participants

We recruited participants via the participant pool of the Department of Experimental Psychology at Ghent University. We excluded two participants before analysis. One person did not understand the instructions of the tasks due to language problems and one person accidently did the focus manipulation and the choice scenario before the assessment of the measures. The final sample of the study consisted of 91 participants (73 female, 17 male, 1 not reported) with an age ranging from 18 to 48 (*M* = 22.80; *SD* = 5.00). Participants received 10 € in return for their participation and were allowed to take home a snack (i.e. Mars, Snickers, banana or apple) after the study was finished.

### Procedure

Upon arrival, participants were seated in separate cubicles and signed an informed consent. We then assessed two explicit measures—that is, tastiness and habit of chocolate and fruit consumption. In order to minimize the influence of carry-over effects of the explicit measures on choice we assessed the explicit measures always first. Afterwards, we assessed three different implicit measures: the manikin task, the affective priming task and the ID-EAST. The order of the implicit measures was counterbalanced across participants. To complete all three implicit measures, participants needed approximately 40 minutes. After the assessment of all measures we induced in half of the participants an affective focus and in the other half of participants a cognitive focus. Participants then completed two different choices. First, they hypothetically chose on paper five food options (i.e. Mars, Snickers, banana or apple). Second, they actually chose one of the chocolate bars or fruits that they were allowed to take home for consumption. Finally, participants were fully debriefed and dismissed.

### Materials and apparatus

For the implicit measures, we took eight fruit pictures that included four different pictures of bananas and four different pictures of apples. Chocolate pictures included eight pictures of two different chocolate brands: four pictures of “Mars” and four pictures of “Snickers”. For the affective priming task and the ID-EAST we selected from the International Affective Picture System [IAPS; 49] in addition to the food pictures, pictures that differed in valence, but were equal in arousal. That is, we selected eight negative pictures (i.e. 6200, 9325, 6563, 3185, 2770, 2900.1, 9341, 9584) and eight positive pictures (i.e. 2216, 2209, 8186, 5825, 4610, 5210, 2070, 8162). All pictures had a size of 1024 x 768 pixels.

We conducted the experiment on Asus Eee PC 1215N laptops with Windows 7 as the system software. Instructions and tasks were presented on external 17-inch Dell monitors. Participants viewed the screen from a distance of approximately 45 cm and provided responses on a “QUERTY” keyboard. The implicit tasks were programmed using Tscope5 software [50].

### Measures

#### Self reported tastiness rating

Participants were asked to indicate on 7-point scales ranging from 1 (*not at all*) to 7 (*very much*) how several adjectives applied to Mars, Snickers, banana, and apple. Half of the items were positive (i.e., tasty, pleasant, delicious), whereas the other half of the items was negative (i.e., unsavory, disgusting, unpleasant). To build a relative measure for the self-reported tastiness rating, we first subtracted separately for chocolate and fruit the negative items from the positive items. We then computed the difference between these two scales such that high values indicate a positive taste of chocolate, compared to fruit.

#### Self reported habit

On 7-point scales ranging from 1 (*very rarely*) to 7 (*very often*) participants indicated how often they eat each of the different food options (i.e., Mars, Snickers, banana, apple). To compute a relative habit score, we subtracted the mean of the fruit scales from the mean of the chocolate scales such that high values indicate higher habit of chocolate, compared to fruit.

#### Manikin task

We used the same food pictures as in all other implicit tasks. The manikin was a simple drawing of a person. Participants were instructed to imagine being the manikin and to move with the manikin by pressing the up and down keys of the keyboard. Following the procedure used by De Houwer et al. [16], a trial started with the manikin appearing in either the upper or the lower half of the screen. After 750 ms, a picture of a food option was presented in the center of the screen. Then, participants had to press the appropriate key three times in order to move the manikin up or down the screen. Depending on the initial position of the figure and the movement direction, the figure stopped either at the edge of the screen or close to the picture. The screen turned black 100 ms after the third key press. If participants made an incorrect response, an error message appeared immediately after the first key press for 500 ms. The time between the onset of the stimulus and the first key press served as the dependent variable.

Participants completed two experimental blocks. In one block, participants were instructed to move the manikin as quickly and accurately as possible toward chocolate pictures and away from fruit pictures. In the other block, participants had to move the manikin away from chocolate pictures and toward fruit pictures. The order of the blocks was counterbalanced across participants and each block contained 80 trials. Each block was preceded by 16 practice trials. The manikin appeared equally often in the top half and the bottom half of the screen.

In line with the suggestion from Krieglmeyer et al. [26] we discarded trials with incorrect responses (7%) and responses with latencies below 150 ms and above 1,500 ms (l.1% of the correct responses) to prepare data for analysis. In addition, the scores of three participants were discarded, because of a defective number of trials suggesting that the program crashed or that the participants restarted the program themselves after a couple of trials. We log-transformed all latencies. To compute a total manikin score, for each food category (i.e. chocolate and fruit), we first subtracted mean approach scores from mean avoidance scores. Then, we subtracted the fruit score from the chocolate score such as high values indicate a relative strong approach (as compared to avoidance) tendency toward chocolate (as compared to fruit).

#### Affective priming

We adapted an affective priming task that was already used by Degner and Wentura [48]. As primes, we used the same fruit and chocolate pictures that we used in the other implicit tasks. The target pictures consisted of positive and negative valenced pictures. The task included one training block containing 64 trials in which only the valenced pictures were presented and a second training block of 32 trials in which the fruit pictures were included as trials and one experimental block containing 160 trials. Every trial began with the presentation of a fixation cross for 100 ms. Then, in all blocks except the first training block, a prime stimulus (i.e., chocolate or fruit picture) was presented for 300 ms. After a blank screen of 100 ms a positive or negative picture appeared. Participants’ task was to quickly and accurately categorize these target pictures according to their valence by means of a key press (Q = negative vs. P = positive). Participants received for 500 ms instantaneous accuracy feedback after each trial. Within the experimental block, each prime was presented five times in each target condition. To prepare data for analyses, we first deleted all trials with no response (1.1%) and erroneous trials (5.5%). In addition, the score of one participant, who responded with the wrong keys, was deleted. Similarly as in the Manikin task, we deleted responses with latencies below 150 ms and above 1,500 ms (0.2% of the correct responses) and then log-transformed all latencies. To prepare data for analysis, we subtracted the difference between the mean latency of fruit/negative trials and fruit/positive trials from the difference between the mean latency of chocolate/negative trials and chocolate/positive trials. Thus, positive affective priming scores indicate a relative preference for chocolate over fruit.

### ID-EAST

We used an adapted version of the picture-based ID-EAST that was introduced by Huijding and De Jong [51]. Target stimuli in the ID-EAST were the same chocolate and fruit pictures that we used within the other implicit measures. Positive and negative attribute stimuli were the same stimuli that we used in the affective priming task. In contrast to the other implicit tasks, we used two versions of food pictures. In one version the frame of the food pictures was colored blue and in the other version the frame was colored green. Participants were told that if the picture was not a food item, the meaning of the picture was important. That is, they were instructed to press the positive key (Q) for positive pictures, and the negative key (P) for negative pictures. Participants were also informed that some pictures would be food items. If one of these food items appeared in a green frame, they had to press the positive key; if it was presented in a blue frame, they had to press the negative key. Participants were instructed to respond as fast as possible and as accurately as possible.

The experiment started with a target practice block of 32 trials in which each of the food pictures was presented once in a green frame, and once in a blue frame. This block was followed by an attribute practice block of 32 trials in which the attribute pictures were each presented two times. Finally, participants were presented with four mixed experimental blocks of 32 trials each. In every experimental block, each of the 16 attribute pictures was presented once. Each of the 8 food pictures was presented once in a blue frame and once in a green frame. In all blocks, stimuli were presented in random order.

Each trial started with a fixation cross for 500 ms, followed by the stimulus, which stayed on the screen for 2500 ms. If participants made an incorrect response, a error feedback appeared on the screen for 500 ms. In order to prepare data for analysis we followed the recommendations of De Houwer and colleagues [7,12]. That is, we first discharged trials of incorrect responses (9.2%). Furthermore, reaction times shorter than 300 ms (0.1%) or longer than 3000 ms (0%) were recoded to 300 and 3000 ms, respectively. Then, we log-transformed all latencies. To compute an EAST total score, we first calculated an EAST score for each food category (i.e., chocolate and fruit) by subtracting positive responses from negative responses. Then, we subtracted the fruit EAST score from the chocolate EAST score such that high values indicate a relative preference for chocolate over fruit.

#### Induction of affective or cognitive focus and choice tasks

Before participants chose a snack, they were randomly assigned to one of two focus conditions. The same focus manipulation was already used by Scarabis et al. [37]. In the affective focus condition participants imagined a situation in which they would really enjoy eating a bar of chocolate or fruit and were asked to think about which of the two snacks would make their mouths water more. Furthermore, they were asked to close their eyes and to take a moment to imagine the taste of chocolate or fruit. Participants in the cognitive focus condition were also instructed to think about their preference for one of the snacks, but in contrast to the affective focus condition, they were asked to carefully analyze their reasons and to think of at least five arguments concerning the snacks. The processing time for both conditions was limited to 3 minutes. After participants completed the manipulation, we administered two different choices.

#### Hypothetical choice

First, we told participants to imagine that they could take home 5 food items. Participants then indicated on a paper questionnaire how many of each food option (i.e. Mars, Snickers, apple, banana) they would choose. Participants were allowed to chose 5 times the same food or to chose different food items. To prepare data for analysis we subtracted the amount of chosen fruits from the amount of chosen chocolate bars. High values indicate a relative preference for chocolate as compared to fruit.

#### Actual choice

Second, the experimenter gave participants a plate with a lid on it. On the plate the same snacks that we presented in the implicit measures (i.e. Mars, Snickers, apple, banana) were arranged. When the experimenter lifted the lid, participants were asked to grab one of the snacks. After participants left the lab, the experimenter recorded their choice (0 = fruit; 1 = chocolate).

## Results

### Correlation among measures

To test how the different measures are related to each other, in a first analysis, we investigated the correlation among all applied explicit and implicit measures. As can be seen in Table 1 the two explicit measures (i.e. self-reported tastiness rating and habit) highly correlate with each other, *r* = .65, *p* < .001. Also, the manikin task correlates with the EAST, *r* = .25, *p* = .019, and marginally with self-reported habit, *r* = .20, *p* = .061. All other measures did not correlate with each other.

**Table 1:** Intercorrelations between all explicit measures (tastiness rating, habit) and implicit measures (manikin, affective priming, EAST).

	1.	2.	3.	4.	5.
1. Tastiness rating	-	.65**	.08	.03	-.03
2. Habit		-	.20^†^	.00	.15
3. Manikin			-	.12	.25*
4. Affective priming				-	.07
5. ID-EAST					-

^†^*p* < .07

*p < .05.

** *p* < .001.

### Prediction of choice

To test whether the administered measures predict choice, we ran two regression analyses for each measure. First, we applied multiple regression analyses in order to predict the hypothetical choice on paper. In line with Aiken and West [52] we first z-standardized all continuous variables. We entered the dummy-coded focus manipulation (0 = affective, 1 = cognitive), each measure, and the interaction between the manipulation and each measure as predictors. Second, we ran logistic regression analysis with the MODPROPE macro [53] in order to predict actual choice. We entered the different measures (z-standardized) as predictor and the dummy-coded focus manipulation (0 = affective, 1 = cognitive) as moderator into the equation.

#### Self-reported tastiness rating

In a first analysis we investigated the predictive validity of the self-reported tastiness rating on the hypothetical choice. The results of the regression yielded a significant main effect of tastiness rating, *β* = .66, *t* = 7.94, *p <* .001, indicating that the tastiness rating predicted choice across both focus manipulations. Neither the main effect for the focus manipulation, nor the interaction between the focus manipulation and the tastiness rating were significant, *β*s < .08, *t*s < .70, *p*s > .48.

In a second analysis, we ran logistic regression analysis with actual choice as dependent variable, the tastiness rating as predictor and the manipulation as moderator. Again, the main effect for the tastiness rating was significant, *b* = .86, *SE* = .40, *p =* .033, indicating that the tastiness rating predicted choice across both focus manipulations. Neither the main effect for the manipulation, nor the interaction between the focus manipulation nor the tastiness rating were significant, *p*s > .60.

#### Self-reported habit

To test the predictive validity of the self-reported habit score, we ran the same regression analyses. For the hypothetical choice the regression yielded a significant main effect for the habit score (*β* = .57, *t* = 6.30, *p <* .001) indicating that the habit rating predicted choice across both focus manipulations. The main effect for the manipulation and the interaction between habit and manipulation was not significant, *β*s < .07, *t*s < .63, *p*s > .53.

When predicting actual choice, the logistic regression analysis yielded a marginally significant main effect for the habit score, *b* = .59, *SE* = .31, *p =* .055. Neither the main effect of the manipulation nor the interaction between habit and the manipulation was significant, *p*s > .18.

#### Manikin task

When analyzing the manikin task, the results indicate that the manikin task was neither a significant predictor of hypothetical choice (*β* = .16, *t* = 1.40, *p =* .164), nor for actual choice (*b* = .46, *SE* = .33 *p* = .17). For both dependent variables, neither the focus manipulation, nor the interaction between the manikin task and the focus manipulation were significant, *p*s > .13.

#### Affective priming

The same analyses for the affective priming as predictor yielded no significant results at all, *p*s > .16. The only trend that could be detected was a marginal significant main effect for the affective priming task for the prediction of hypothetical choice, *β* = .18, *t* = 1.70 *p =* .094.

#### ID-EAST

For the ID-EAST as predictor, the regressions did not yield any significant effects nor trends, *p*s > .39.

#### Incremental predictive validity

To test which of the measures contribute to the prediction of choice independently from each other, we, first, computed a step-wise multiple regression analysis in order to predict hypothetical choice. As predictors, we entered the self-reported tastiness rating, self-reported habit, the manikin score, the affective priming score, the ID-EAST, the dummy-coded focus manipulation (0 = affective, 1 = cognitive), as well as all interactions between the focus manipulation and the different measures. The regression suggests a model with self-reported tastiness (*β* = .67, *t* = 8.54 *p <* .001) and the affective priming score (*β* = .17, *t* = 2.20 *p =* .031) as predictors. Adding any of the other predictors does not increase the predictive validity of the hypothetical choice.

For actual choice, we ran a logistic regression with the same predictors. The results indicate that the best predictor is self-reported habit, *b* = .87, *SE* = .28 *p =* .002. Adding any other predictor does not improve the prediction.

### Reliability of implicit measures

In a further analysis, we tested the split-half reliability of the implicit measures. First, we computed the manikin score, the affective priming score and the ID-EAST score separately for even trials and for odd trials. Second, we computed the Spearman-Brown coefficient in order to test the reliability of the implicit measures. The results yielded ρ* = .90 for the manikin task indicating high reliability. However, the Spearmen-Brown coefficient for the affective priming task (ρ* = -.11) and the ID-EAST (ρ* = -.02) were negative indicating non-reliability.

## Discussion

Past research suggests that implicit measures are good predictors of behavior. Thereby, it has been demonstrated that implicit measures are especially good predictors of impulsive and affectively driven behavior [17,32–37]. However, to the best of our knowledge, no study actually tested which implicit measure best predicts behavior. Therefore, in the present study we applied three implicit measures (i.e., manikin task, affective priming, ID-EAST) and tested their predictive validity of either affectively or cognitively driven consumption behavior. To cross-validate the predictive validity of the implicit measures we assessed in addition two explicit measures (i.e. self-reported habit and tastiness).

Correlational analyses among the implicit measures show that the manikin task correlates with the ID-EAST, but the affective priming task does not correlate with any of the implicit measures. This indicates that the manikin task and the ID-EAST share variance independent from the affective priming task. This might be due to similarities and differences between the tasks’ characteristics. In the manikin task as well as in the ID-EAST, participants respond to the target stimuli (i.e. chocolate and fruit) by the selection of two key-presses that are related to valence. However, in the affective priming task, participants respond not at all to the target category. Here, the target stimuli are the primes that precede either positive or negative stimuli and participants respond to the positive and negative stimuli, but not to the target stimuli itself. Thus, it might well be that this difference in the response accounts for the lack of correlation among the measures.

When testing the association of the implicit measures with consumption behavior, we found that the manikin task marginally correlates with self-reported habit. All other implicit measures did not correlate with any self-reported measure. When testing the predictive validity of all measures, the results indicate that the two explicit measures (i.e. self-reported tastiness and habit), but not the implicit measures (i.e., manikin task, affective priming, ID-EAST), predict consumer choice across both foci very well. Further regression analyses testing the incremental validity of the different tasks indicated that the explicit measures were the best predictors of choice. When adding the implicit measures into the regression equations only the affective priming task could add some variance in explaining consumer choice. This overall pattern suggest that explicit measures are better predictors of consumer choice than implicit measures.

An interesting question is why the implicit measures showed rather low predictive validity. First, a possible reason may lay in the reliability of the tasks. Psychometric theory has shown that unreliable tasks are less likely to correlate with other tasks (cf., [54,55]). Both, the ID-EAST and the affective priming task were unreliable and may, thus, suffer from lack in predictive validity. In contrast, the manikin task showed high reliability, but did not show high predictive validity for consumer choice. A plausible reason may lay in our chosen consumer products (fruit vs. chocolate), which do not very much differ in valence. While past research has shown that approach-avoidance tasks are sensitive for stimuli differing in valence [13–16,18,20], our research indicates that such tasks might be less sensitive for stimuli that differ to a smaller degree in valence. Second, another reason for the bad predictive validity might be lack of power. In fact, it could well be that with more participants in our sample some correlations would have become significant. However, even if this would be the case, it would indicate that the predictive validity of the implicit measures is rather low and not as high as the predictive validity of the explicit measures. Third, we have to mention possible demand effects that could explain the good predictive validity of explicit measures in dispense of the implicit measures. That is, during choice, participants may still have remembered their explicit ratings and then went along with their previous indication. Unfortunately, we cannot rule out this alternative explanation although we tried to reduce potential demand effects by assessing the explicit measures as the first measures in the hope that the impact of the assessment of choice would be minimized by such a procedure. However, we cannot rule out the potential influence it may had. Fourth, it could be that the large number of trials across all the implicit measures induced noise into the data, because participants started habituating to the stimuli. In order to deal with this issue we recommend for future research between-subject designs in which only one implicit measure per between-factor is applied.

Another limitation of our experiment concerns the focus manipulation. While other studies found that by applying the same manipulation implicit measures better predicted choice in the affective focus condition than in the cognitive focus condition [17,37,38], we were not able to replicate such a finding. There might be a couple of reasons why this is the case. First, past research used different tasks in order to predict behavior than we did. Genschow and colleagues [17] found that another approach task, but not a similar affective priming task, predicted consumer choice in the affective, but not in the cognitive focus condition. Scarabis et al. [37] as well as Smith and Nosek [38] used an IAT and found increased predictive validity in the affective, as compared to the cognitive condition. It might well be that our applied tasks are not as much dependent on the mode of choice as the tasks that have been assessed by other researchers. However, future research may test this interpretation in more detail by applying other manipulations and by comparing other tasks with each other. Second, it might be that due to the fact that participants saw the stimuli within all tasks many times, they started thinking about the stimuli already before the manipulation, which could have diminished the effectiveness of the affective focus manipulation. In addition, for both manipulations we gave participants 3 minutes time. Potentially, 3 minutes could have been too long for the affective focus manipulation, so participants started elaborating on the food stimuli, which in turn might have diminished the affective manipulation even more strongly. Third, the affective focus manipulation might have manipulated different forms of affect. That is, research has shown that affect is related to fast and intuitive decisions (e.g., [56,57]), but also to emotions (e.g., [58]). These two different forms of affect might not influence choices in the same direction and might, thus, have, diminished our manipulation. Future research should consider disentangling different forms of affect in order to establish a more precise manipulation. Fourth, it might be that our manipulation actually did not work. That is, the manipulation failed in inducing an affective and cognitive focus. Although we followed the procedure of other research, we cannot rule out this alternative explanation. One may argue that a manipulation check would have allowed gaining knowledge about the effectiveness of the manipulation. However, to the best of our knowledge, such a manipulation check does not exist in the literature and has, thus, never been previously applied.

## References

[1] Greenwald AG, Banaji MR (1995) Implicit social cognition: attitudes, self-esteem, and stereotypes. Psychological review 102: 4–27. 7878162 10.1037/0033-295x.102.1.4

[2] Wilson TD, Lindsey S, Schooler TY (2000) A model of dual attitudes. Psychological review 107: 101–126. 10687404 10.1037/0033-295x.107.1.101

[3] De Houwer J (2006) What are implicit measures and why are we using them. The handbook of implicit cognition and addiction: 11–28.

[4] Fazio RH, Jackson JR, Dunton BC, Williams CJ (1995) Variability in automatic activation as an unobtrusive measure of racial attitudes: a bona fide pipeline? Journal of personality and social psychology 69: 1013–1027. 8531054 10.1037//0022-3514.69.6.1013

[5] Fazio RH, Sanbonmatsu DM, Powell MC, Kardes FR (1986) On the automatic activation of attitudes. Journal of Personality and Social Psychology 50: 229–238. 3701576 10.1037//0022-3514.50.2.229

[6] Greenwald AG, McGhee DE, Schwartz JL (1998) Measuring individual differences in implicit cognition: the implicit association test. Journal of personality and social psychology 74: 1464–1480. 9654756 10.1037//0022-3514.74.6.1464

[7] De Houwer J, De Bruycker E (2007) The identification-EAST as a valid measure of implicit attitudes toward alcohol-related stimuli. Journal of Behavior Therapy and Experimental Psychiatry 38: 133–143. doi: 10.1016/j.jbtep.2006.10.004 17109815 10.1016/j.jbtep.2006.10.004

[8] De Houwer J, Teige-Mocigemba S, Spruyt A, Moors A (2009) Implicit measures: A normative analysis and review. Psychological bulletin 135: 347–368. doi: 10.1037/a0014211 19379018 10.1037/a0014211

[9] De Houwer J, De Bruycker E (2007) The implicit association test outperforms the extrinsic affective Simon task as an implicit measure of inter-individual differences in attitudes. British Journal of Social Psychology 46: 401–421. doi: 10.1348/014466606X130346 17565789 10.1348/014466606X130346

[10] Schmukle SC, Egloff B (2006) Assessing anxiety with extrinsic Simon tasks. Experimental Psychology 53: 149–160. doi: 10.1027/1618-3169.53.2.149 16909940 10.1027/1618-3169.53.2.149

[11] Teige S, Schnabel K, Banse R, Asendorpf JB (2004) Assessment of multiple implicit self‐concept dimensions using the Extrinsic Affective Simon Task (EAST). European Journal of Personality 18: 495–520.

[12] De Houwer J (2003) The extrinsic affective Simon task. Experimental psychology 50: 77–85. doi: 10.1026//1618-3169.50.2.77 12693192 10.1026//1618-3169.50.2.77

[13] Bamford S, Ward R (2008) Predispositions to approach and avoid are contextually sensitive and goal dependent. Emotion 8: 174–183. doi: 10.1037/1528-3542.8.2.174 18410191 10.1037/1528-3542.8.2.174

[14] Brendl CM, Markman AB, Messner C (2005) Indirectly measuring evaluations of several attitude objects in relation to a neutral reference point. Journal of Experimental Social Psychology 41: 346–368.

[15] Chen M, Bargh JA (1999) Consequences of automatic evaluation: Immediate behavioral predispositions to approach or avoid the stimulus. Personality and Social Psychology Bulletin 25: 215–224.

[16] De Houwer J, Crombez G, Baeyens F, Hermans D (2001) On the generality of the affective Simon effect. Cognition & Emotion 15: 189–206.

[17] Genschow O, Florack A, Chib VS, Shimojo S, Scarabis M, Wänke M (2013) Reaching for the (product) stars: Measuring recognition and approach speed to get insights into consumer choice. Basic and Applied Social Psychology 35: 298–315.

[18] van Dantzig S, Pecher D, Zwaan RA (2008) Approach and avoidance as action effects. The Quarterly Journal of Experimental Psychology 61: 1298–1306. doi: 10.1080/17470210802027987 19086189 10.1080/17470210802027987

[19] Elliot AJ (2008) Handbook of approach and avoidance motivation New York: Psychology Press.

[20] Genschow O, Florack A, Wänke M (2014) Recognition and approach responses toward threatening objects. Social Psychology: 904–907.

[21] Eder AB, Rothermund K (2008) When do motor behaviors (mis) match affective stimuli? An evaluative coding view of approach and avoidance reactions. Journal of Experimental Psychology: General 137: 262–281. 18473659 10.1037/0096-3445.137.2.262

[22] Morange F, Bloch H (1996) Lateralization of the approach movement and the prehension movement in infants from 4 to 7 months. Early Development and Parenting 5: 81–92.

[23] Seibt B, Neumann R, Nussinson R, Strack F (2008) Movement direction or change in distance? Self-and object-related approach-avoidance motions. Journal of Experimental Social Psychology 44: 713–720.

[24] von Hofsten C, Rönnqvist L (1988) Preparation for grasping an object: A developmental study. Journal of Experimental Psychology: Human Perception and Performance 14: 610–621. 2974872 10.1037//0096-1523.14.4.610

[25] Krieglmeyer R, Deutsch R (2010) Comparing measures of approach-avoidance behaviour: The manikin task vs. two versions of the joystick task. Cognition and Emotion 24: 810–828.

[26] Krieglmeyer R, Deutsch R, De Houwer J, De Raedt R (2010) Being moved valence activates approach-avoidance behavior independently of evaluation and approach-avoidance intentions. Psychological Science 21: 607–613. doi: 10.1177/0956797610365131 20424109 10.1177/0956797610365131

[27] Millar MG, Tesser A (1986) Effects of affective and cognitive focus on the attitude–behavior relation. Journal of Personality and Social Psychology 51: 270–276.

[28] Millar MG, Tesser A (1992) The role of beliefs and feelings in guiding behavior: The mismatch model. The construction of social judgments: 277–300.

[29] Zanna MP, Rempel JK (1988) Attitudes: A new look at an old concept In: Bar-Tal D, Kruglanski AW, editors. The social psychology of knowledge. Cambridge, England: Cambridge University Press pp. 315–334.

[30] Asendorpf JB, Banse R, Mücke D (2002) Double dissociation between implicit and explicit personality self-concept: the case of shy behavior. Journal of personality and social psychology 83: 380–393. 12150235

[31] Dovidio JF, Kawakami K, Johnson C, Johnson B, Howard A (1997) On the nature of prejudice: Automatic and controlled processes. Journal of experimental social psychology 33: 510–540.

[32] Florack A, Friese M, Scarabis M (2010) Regulatory focus and reliance on implicit preferences in consumption contexts. Journal of Consumer Psychology 20: 193–204.

[33] Friese M, Hofmann W, Wänke M (2008) When impulses take over: Moderated predictive validity of explicit and implicit attitude measures in predicting food choice and consumption behaviour. British Journal of Social Psychology 47: 397–419. doi: 10.1348/014466607X241540 17880753 10.1348/014466607X241540

[34] Friese M, Hofmann W, Wänke M (2009) The impulsive consumer: Predicting consumer behavior with implicit reaction time measures In: Wänke M, editor. Social psychology of consumer behavior. New York: Psychology Press pp. 335–364.

[35] Friese M, Wänke M, Plessner H (2006) Implicit consumer preferences and their influence on product choice. Psychology & Marketing 23: 727–740.

[36] Hofmann W, Gschwendner T, Nosek BA, Schmitt M (2005) What moderates implicit—explicit consistency? European Review of Social Psychology 16: 335–390.

[37] Scarabis M, Florack A, Gosejohann S (2006) When consumers follow their feelings: The impact of affective or cognitive focus on the basis of consumers' choice. Psychology & Marketing 23: 1015–1034.

[38] Smith CT, Nosek BA (2011) Affective focus increases the concordance between implicit and explicit attitudes. Social Psychology 42: 300–313.

[39] Bar-Anan Y, Nosek BA (2014) A comparative investigation of seven indirect attitude measures. Behavior research methods 46: 668–688. doi: 10.3758/s13428-013-0410-6 24234338 10.3758/s13428-013-0410-6

[40] Sriram N, Greenwald AG (2009) The brief implicit association test. Experimental psychology 56: 283–294. doi: 10.1027/1618-3169.56.4.283 19439401 10.1027/1618-3169.56.4.283

[41] Karpinski A, Steinman RB (2006) The single category implicit association test as a measure of implicit social cognition. Journal of personality and social psychology 91: 16 doi: 10.1037/0022-3514.91.1.16 16834477 10.1037/0022-3514.91.1.16

[42] Nosek BA, Banaji MR (2001) The go/no-go association task. Social cognition 19: 625–666.

[43] Payne BK, Cheng CM, Govorun O, Stewart BD (2005) An inkblot for attitudes: affect misattribution as implicit measurement. Journal of personality and social psychology 89: 277–293. doi: 10.1037/0022-3514.89.3.277 16248714 10.1037/0022-3514.89.3.277

[44] Bar-Anan Y, Nosek BA, Vianello M (2009) The sorting paired features task: A measure of association strengths. Experimental psychology 56: 329–343. doi: 10.1027/1618-3169.56.5.329 19447749 10.1027/1618-3169.56.5.329

[45] Brunel FF, Tietje BC, Greenwald AG (2004) Is the implicit association test a valid and valuable measure of implicit consumer social cognition? Journal of Consumer Psychology 14: 385–404.

[46] Greenwald AG, Poehlman TA, Uhlmann EL, Banaji MR (2009) Understanding and using the Implicit Association Test: III. Meta-analysis of predictive validity. Journal of personality and social psychology 97: 17–41. doi: 10.1037/a0015575 19586237 10.1037/a0015575

[47] Maison D, Greenwald AG, Bruin RH (2004) Predictive validity of the Implicit Association Test in studies of brands, consumer attitudes, and behavior. Journal of consumer psychology 14: 405–415.

[48] Degner J, Wentura D (2010) Automatic prejudice in childhood and early adolescence. Journal of personality and social psychology 98: 356–374. doi: 10.1037/a0017993 20175618 10.1037/a0017993

[49] Lang PJ, Bradley MM, Cuthbert BN (2008) International affective picture system (IAPS): Affective ratings of pictures and instruction manual. Technical report A-8.

[50] Stevens M, Lammertyn J, Verbruggen F, Vandierendonck A (2006) Tscope: AC library for programming cognitive experiments on the MS Windows platform. Behavior Research Methods 38: 280–286. 16956104 10.3758/bf03192779

[51] Huijding J, De Jong P (2005) A Modified Extrinsic Affective Simon Task (EAST) to assess the affective value of pictorial stimuli: No influence of age and educational level. Psychologica Belgica 45: 241–255.

[52] Aiken LS, West SG (1996) Multiple Regression testing and interpreting interactions London: Sage Publications.

[53] Hayes AF, Matthes J (2009) Computational procedures for probing interactions in OLS and logistic regression: SPSS and SAS implementations. Behavior research methods 41: 924–936. doi: 10.3758/BRM.41.3.924 19587209 10.3758/BRM.41.3.924

[54] Crocker L, Algina J (1986) Introduction to classical and modern test theory Fort Worth, TX: Holt, Rinehart, & Winston.

[55] Cronbach LJ (1990) Essentials of psychological testing (5th ed.). New York: Harper & Row.

[56] Slovic P, Peters E, Finucane ML, MacGregor DG (2005) Affect, risk, and decision making. Health psychology 24: 35–40. 10.1037/0278-6133.24.4.S35 16045417

[57] Topolinski S, Strack F (2009) The analysis of intuition: Processing fluency and affect in judgements of semantic coherence. Cognition and Emotion 23: 1465–1503.

[58] Schulz R (1985) Emotion and affect In: Birren JE, Schaie KW, editors. Handbook of the psychology of aging, 2nd ed. New York, NY: Van Nostrand Reinhold Co. pp. 931 pp.

